# Domestic Correspondence

**Published:** 1890-01

**Authors:** 


### Domestic Correspondence.

To the Editor:

*American Academy of Dental Science.*—The twenty-second annual meeting of the American Academy of Dental Science was held at Young's Hotel, in Boston, on Wednesday, November 13, 1889. There was a very good attendance of members present, and the chair was occupied by the president, Dr. Cecil P. Wilson, of Boston. He welcomed the members to the hospitalities of this twenty-second anniversary, and spoke of the pleasures and benefits derived from gatherings like this, of earnest professional gentlemen united together for the public good and for the promotion of the best interests of their chosen profession, and he congratulated them upon the prosperity and progress of the Academy. After listening to the annual reports, and other routine business having been transacted, the following officers were elected for the ensuing year: President, Dr. F. N. Seabury, of Providence, R. I.; Vice-President, Dr. C. A. Brackett, of Newport, R. I.; Corresponding Secretary, Dr. E. N. Harris, of Boston; Recording Secretary, Dr. V. C. Pond, of Boston; Treasurer, Dr. E. C. Briggs, of Boston; Librarian, Dr. Charles Wilson, of Boston.

*Executive Committee.*—Dr. E. H. Smith, of Boston; Dr. Thomas Fillebrown, of Portland, Maine; and Dr. F. E. Banfield, of Boston.

Dr. W. N. Potter, of Boston, was appointed editor of the Academy proceedings for the ensuing year. Dr. Harris, corresponding secretary of the Academy from 1867 to 1877, by urgent solicitation of the members, consented to accept that position again.

Resolutions of respect to the memory of Dr. F. Searle, of Springfield; Dr. S. P. Stearns, of Boston; and Dr. J. W. Smith, of Newport, members of the Academy who departed this life during the past year, were adopted by a rising vote.

The annual address was delivered by Dr. J. T. Codman, of Boston, taking for his subject "Retrospect and Prospect." He spoke of the conditions of life under which professional men lived seventy-five years ago, and gave a very interesting sketch of their surroundings, and a synopsis of the many improvements made in society and in dentistry since that time.

A vote of thanks was given to Dr. Codman for his address, and a copy requested for publication.

A vote of thanks was also presented to the retiring president, Dr. Wilson, for the able and impartial manner in which he has presided over the Academy during the past two years.

At six o'clock, adjourned to partake of the anniversary dinner and banquet, which was an elegant affair. The newly-elected president, Dr. Seabury, presided, and he thanked the members for the honor conferred upon him, and called upon the venerable Dr. Elisha G. Tucker, who was present, and now in his eighty-second year, for some remarks. Dr. Tucker responded, and expressed his gratification at having had the pleasure and privilege of attending every annual meeting since the Academy was organized, and spoke of the old pioneer dental practitioners of Boston and other parts of our country, who have nearly all passed away. Other pertinent and well-timed remarks were made by many of the members, and everything passed off very happily.

This society was founded in 1867, in Boston, and its career has been one of uninterrupted success, and it has accomplished much for the advancement of dental science and for the elevation of the profession. Monthly meetings are held at the rooms of the Boston Medical Library Association, 19 Boylston Place, which are well attended and replete with interest. At the last meeting, held December 4, papers were read by Dr. D. W. Fellows, of Portland, upon "Fracture of the Jaws," and by Dr. J. E. Waitt, of Boston, on "A Rapid Method of Inserting and Finishing Contour Fillings without a Matrix." Each paper was followed by a discussion on the subject presented. Dr. F. G. Eddy, of Providence, exhibited a new mouth-piece for saliva-ejectors, and Dr. Waitt exhibited a nitrous-oxide light for photo-microscopic purposes. George T. Baker, D.D.S., of Boston, and William S. Sherman, M.D., D.D.S., of Newport, were elected associate members, and W. W. H. Thackston, M.D., D.D.S., of Farmville, Virginia, was elected an honorary member.

EDWARD N. HARRIS,
*Corresponding Secretary.*

366 COLUMBUS AVENUE, BOSTON.

---

TO THE EDITOR:

I think you would do the profession a favor if you called attention to the Perry Separators editorially. They have been written up by Dr. Perry very fully, but I do not think that they have been appreciated by those who have not tried them as they deserve to be. Among my friends I find few who have used them. They are expensive; but from two months' use I am free to say that, to a dentist who has even a moderate practice, they would pay for themselves in the increased work he can do if he had to get a full set every three months, to say nothing of the better work that can be done with them, and the comfort they are to both patient and operator. If used with judgment, they give at once sufficient working space, and cause, practically, no pain. I have repeatedly separated the six front teeth, opening and examining each space at one sitting, that too for nervous, "skittish" children, without objections being made by the patient, and in no case have I seen any injury done, or their use followed by any unpleasant after-effect. I can readily appreciate how their use, in the hands of one who was anxious to see how much space he could make, or one who forgets that patients have feelings, would be very painful, and that serious and permanent injury might follow. They were, I venture to say, not invented to be so used. There is a little pain at times, in separating the molar teeth, but if they are opened slowly it is very bearable and soon over.

There is need for a dam clamp that can be used without being in the way of the separator. With all that I have or have seen, the clamp must be put one tooth back of the separator or the dam be held by a ligature. Either expedient is at times very inconvenient, and in some cases either the dam or the separator must be dispensed with.

WILLIAM H. TRUEMAN.

PHILADELPHIA.

---

TO THE EDITOR:

Composition and process for tempering instruments: rosin, 7½ parts; whale oil, 1½ parts; pulv. charcoal, ½ part.

Directions for use: the instruments should be dipped in the mixture same as in water, then polish and draw the temper as usual. Small instruments should be dipped but once, larger ones two or three times. For engine and other small drills, which I want *very* hard, I heat to a bright red and dip in just the drill point, and do not draw the temper. In dipping for this last work dip the point straight down, not obliquely.

WILLIAM STEELE, D.D.S.

FOREST CITY, IOWA.

